# The Effect of Ursodeoxycholic Acid (UDCA) on Serum Expression of miR-34a and miR-506 in Patients with Chronic Cholestatic Liver Diseases

**DOI:** 10.3390/cells14151137

**Published:** 2025-07-23

**Authors:** Eliza Cielica, Alicja Łaba, Piotr Milkiewicz, Beata Kruk, Agnieszka Kempinska-Podhorodecka, Patrycja Kłos, Pedro M. Rodrigues, Beatriz Val, Maria J. Perugorria, Jesus M. Banales, Malgorzata Milkiewicz

**Affiliations:** 1 Department of Medical Biology, Pomeranian Medical University, 70-111 Szczecin, Poland; eliza.cielica@o2.pl (E.C.); alicja.laba@pum.edu.pl (A.Ł.); agnieszka.kempinska.podhorodecka@pum.edu.pl (A.K.-P.); 2 Liver and Internal Medicine Unit, Department of General, Transplant and Liver Surgery, Medical University of Warsaw, 02-097 Warsaw, Poland; p.milkiewicz@wp.pl; 3 Translational Medicine Group, Pomeranian Medical University, 70-204 Szczecin, Poland; 4 Laboratory of Metabolic Liver Diseases, Department of General, Transplant and Liver Surgery, Medical University of Warsaw, 02-097 Warsaw, Poland; beata.kruk@wum.edu.pl; 5 Department of Biochemistry and Medical Chemistry, Pomeranian Medical University in Szczecin, 70-204 Szczecin, Poland; patrycja.klos@pum.edu.pl; 6 Department of Liver and Gastrointestinal Diseases, Biogipuzkoa Health Research Institute, Donostia University Hospital, University of the Basque Country (UPV/EHU), 20014 Donostia-San Sebastian, Spain; pedromiguel.rodriguesvieira@bio-gipuzkoa.eus (P.M.R.); beatriz.coelhodoval@bio-gipuzkoa.eus (B.V.); majesus.perugorriamontiel@bio-gipuzkoa.eus (M.J.P.); jesusmaria.banalesasurmendi@bio-gipuzkoa.eus (J.M.B.); 7 National Institute for the Study of Liver and Gastrointestinal Diseases (CIBERehd, “Instituto de Salud Carlos III”), 28220 Madrid, Spain; 8 Ikerbasque, Basque Foundation for Science, 48013 Bilbao, Spain; 9 Department of Medicine, Faculty of Medicine and Nursing, University of the Basque Country (UPV/EHU), 20014 Donostia-San Sebastian, Spain; 10 Department of Biochemistry and Genetics, School of Sciences, University of Navarra, 31008 Pamplona, Spain

**Keywords:** ursodeoxycholic acid, cholangitis, miR-34a, miR-506, TREM-2

## Abstract

Ursodeoxycholic acid (UDCA) is widely used to treat cholestatic liver diseases such as primary biliary cholangitis (PBC) and primary sclerosing cholangitis (PSC), yet its molecular mechanisms remain unclear. This study investigated the impact of long-term UDCA therapy on circulating levels of the microRNAs miR-34a and miR-506, which are implicated in PBC pathogenesis, and explored associated changes in inflammatory markers and signaling pathways. Serum samples from patients with PBC and PSC were collected before and after UDCA treatment and analyzed for miRNA expression as well as levels of TREM-2 and sCD163. In vitro studies using human cholangiocytes and lipopolysaccharide (LPS) stimulation assessed changes in the expression of miR-34a, TREM-2, and ADAM17. The results showed that the baseline levels of miR-34a and miR-506 were significantly elevated in PBC patients compared to controls and were significantly reduced after UDCA therapy in PBC but not in PSC. UDCA also decreased serum levels of TREM-2 and sCD163. In vitro, it suppressed the LPS-induced expression of miR-34a and ADAM17 while enhancing TREM-2 expression. Single-cell RNA sequencing of liver tissue and immunofluorescence staining confirmed TREM-2 expression in cholangiocytes. These findings suggest that UDCA modulates key inflammatory pathways and miRNAs in PBC, providing mechanistic insights into its therapeutic effect

## 1. Introduction

Cholestatic liver diseases, which include primary biliary cholangitis (PBC) and primary sclerosing cholangitis (PSC) [1,2], are characterized by impaired bile flow and bile acid accumulation. PBC is a rare, chronic disease primarily affecting middle-aged women. The disease leads to inflammation and the selective destruction of small and intermediate intrahepatic bile ducts, which results in liver fibrosis over the long term. The mechanisms leading to the development of PBC are not fully understood. PSC is characterized by inflammation and an abnormal narrowing of the medium and large ducts in the intrahepatic or extrahepatic biliary tree. Ursodeoxycholic acid (UDCA) is the current treatment for cholestatic liver diseases. UDCA, in contrast to the relative toxicity of other bile acids, has well-established therapeutic properties, prevents histological disease progression and improves long-term survival in PBC patients [3,4,5]. However, nearly one-third of patients are non-responders, and the treatment does not relieve disease-associated behavioral symptoms such as fatigue [6]. How UDCA exerts its hepatoprotective effects is not yet fully understood. Although, it has been claimed that UDCA is an important signaling molecule that modulates liver cell apoptosis [7], acts as an antioxidant [7], regulates the expression of inflammatory cytokines, promotes bile excretion and reduces the severity of cell injury [8].

MicroRNAs (miRNAs) are small, noncoding molecules that affect post-transcriptional mRNA stability and suppress the translation of target mRNA. Dysregulation of the miRNA expression profile in the liver and peripheral blood mononuclear cells (PBMCs) of PBC patients has been reported [9,10]. Different miRNAs, including miR-34a, are involved in liver inflammation and fibrosis [11], and serum levels of miRNA-34a correlate with fibrosis, steatosis and inflammatory activity in the livers of patients with hepatitis C virus (HCV) and metabolic dysfunction-associated steatotic liver disease (MASLD) [12]. MiR-34a promotes the expression of transforming growth factor-beta type 1 receptor (TβR1), TGF-β1 and p-SMAD2/3, which are connected to liver fibrosis and inflammatory cytokines such as interleukin-6 (IL-6) and IL-7 [13]. MiR-34a is up-regulated in the PBMCs of PBC patients [13,14]. Another key miRNA that significantly contributes to PBC pathogenesis is miR-506, as its characteristic overexpression in PBC cholangiocytes targets the Cl-/HCO3- exchanger AE2 and causes multiple PBC features [15,16].

Triggering receptor expressed on myeloid cells 2 (TREM-2) is involved in the modulation of inflammation in different inflammatory diseases. It negatively regulates inflammatory responses mediated by toll-like receptors (TLRs) and is up-regulated in various inflammatory diseases [17,18]. TLRs expressed on the membranes of antigen-presenting cells, including Kupffer cells (KCs) in the liver, are important mediators of inflammatory pathways. Gut-derived bacterial products bound in the liver by TLRs contribute to acute and chronic liver diseases [19]. Moreover, KCs produce mediators that induce the profibrotic phenotype of hepatic stellate cells (HSCs) [20]. During biosynthesis and maturation, TREM-2 undergoes ectodomain shedding and intramembranous cleavage. After removal of the ectodomain by proteases of the A disintegrin and metalloprotease (ADAM) family (including ADAM10 and ADAM17), soluble TREM-2 (sTREM-2) is released into extracellular fluids. The anti-inflammatory TREM-2 receptor protects against cholestasis-induced liver injury in patients with PBC and PSC and in mice with cholestatic liver injury [13,21,22]. MiR-34a has been recognized as an inhibitor of TREM-2 [23]. ADAM17 is activated by INF-γ or lipopolysaccharides (LPSs) and is responsible for the release of liver injury mediators, and the inhibition of ADAM17 has an anti-inflammatory outcome in many autoimmunological diseases [24]. UDCA affects ADAM17 activity, which decreases the amount of shed tumor necrosis factor-α (TNF-α) in HepG2 cell lines [25].

Soluble CD163 (sCD163) is a macrophage activation marker, and its level is associated with liver fibrosis/cirrhosis in patients with chronic viral hepatitis (hepatitis B virus and HCV) [26], MASLD and metabolic dysfunction-associated steatohepatitis (MASH) [27,28], alcoholic liver disease [29], and autoimmune hepatitis [30]. The serum concentration of sCD163 correlates with liver disease severity and the response to UDCA treatment in PBC patients [31,32].

In this prospective study, we investigated the effect of UDCA on the serum expression of miR-34a and miR-506 in patients with chronic cholestatic liver diseases—namely PBC and PSC. The observed changes in response to UDCA treatment were confirmed in vitro in normal cholangiocyte cell lines.

## 2. Materials and Methods

### 2.1. Patients

A cohort of Caucasian patients diagnosed with PBC or PSC was recruited at the Liver and Internal Medicine Unit, Medical University of Warsaw, from September 2018 to December 2022. Both diseases were diagnosed according to the European Association for the Study of the Liver [33] criteria. Serum samples were obtained before treatment with UDCA (PBC, *n* = 27; PSC, *n* = 17) at a dose of 13–15 mg/kg body weight for at least 12 months (median duration: 44 months; range: 22–56 months). Serum obtained from the same patient before and after UDCA treatment (PBC *n* = 11 and PSC *n* = 10) allowed for paired-sample testing. Demographic and laboratory data of the analyzed patients are summarized in Table 1. Additionally, serum samples from 25 healthy Caucasian subjects collected at the University of Warsaw served as sex- and age-matched control groups (control for PBC *n* = 16; control for PSC *n* = 9; Table 2). The study protocol was approved by the Ethics Committee of the Pomeranian Medical University (approval number BN-001/43/06, dated 20 April 2006) and conducted in accordance with the Declaration of Helsinki (6th revision, 2008). Written informed consent was obtained from all participants prior to their inclusion in the study. All patients provided written informed consent to participate in the study.

### 2.2. Cell Culture

Three cell lines were used for the in vitro experiments: an NHC cell line, an immortalized SV40-transformed human biliary epithelial cell line (H69) and an immortalized human cholangiocyte cell line overexpressing miR506 (H69miR506). Cells were cultured in media containing Dulbecco’s Modified Eagle medium (DMEM/F12) with glutamax and penicillin–streptomycin, fetal bovine serum H.I., MEM vitamin solution, MEM non essentials Aas, a chemically defined lipid mixture, epidermal growth factor, soybean trypsin inhibitor, insulin transferrin selenium, 3,3′5-triiodo-L-thyronine, dexamethasone, bovine pituitary extract, forskolin and blasticidin. NHCs were pre-exposed to UDCA (100 µM) for 2 h prior to LPS stimulation. Subsequently, the cells were incubated for 24 h with or without LPS (5 µg/mL, Escherichia coli O111:B4, L4391-1MG, Sigma-Aldrich). UDCA remained in the culture medium throughout the entire experiment, including both the pre-incubation phase and the 24-h incubation with or without LPS. All cell culture work was performed under sterile conditions in a laminar flow hood, and cells were incubated at 37 °C and 5% CO_2_.

### 2.3. Single-Cell Sequencing

Single-cell transcriptomic profiles from liver samples of primary biliary cholangitis (PBC) patients and healthy controls were obtained from the Gene Expression Omnibus (GEO) under accession numbers GSE247128 (*n* = 2) and GSE115469 (*n* = 5), respectively [34,35]. Both PBC patients included in the scRNA-seq analysis were female, whereas the control cohort consisted of four male and one female donor. Preprocessing and downstream analyses were conducted using Seurat v5.1.0 in R v4.3.3.

Initial quality control was applied independently to each dataset. For PBC, cells with fewer than 200 detected genes, more than 5000 detected genes or over 25% mitochondrial gene content were excluded. For control samples, the upper gene threshold was set to 6000, with the same cutoffs for minimum gene expression and mitochondrial content. Normalization was performed using the SCTransform pipeline, regressing out mitochondrial content and total RNA counts to minimize technical variability.

Following normalization, 2000 highly variable genes were selected using the FindVariableFeatures() function with the “vst” method. Dimensionality reduction was then performed using Principal Component Analysis (PCA). Based on elbow plot diagnostics, the top 20 principal components (PCs) for PBC and 30 PCs for control samples were retained for downstream tasks. Clustering was conducted using the Leiden algorithm, with optimal resolutions determined empirically (resolution = 0.2 for PBC, 0.4 for controls). UMAP and *t*-SNE embeddings were computed to visualize the unintegrated cluster structures.

The cell type annotation of clusters was based on the expression of canonical marker genes as follows: hepatocytes (HPX, LBP, and SERPINA10); cholangiocytes (KRT19, KRT7, and FXYD2); fibroblasts (COL1A1 and DCN); endothelial cells (FCN2, VWF, and CDH5); dendritic cells (DCs) (IRF8); macrophages (CD68, CD163, and CSFIR); granulocytes (FXYD2 and IRF8); B cells (MS4A1 and CD79A); plasma cells (FCRL5 and IGHM); natural killer (NK) cells (KLRD1 and GNLY); cytotoxic T cells (CD3D and TRAC); helper T cells (IL7R, MAL); and γδ (gd) T cells (TYMS).

Subsequently, the PBC and control Seurat objects were merged. Reciprocal PCA (RPCA) integration was performed using the IntegrateLayers() function to correct for batch effects. The datasets were then joined using JoinLayers() to create a harmonized object. Post-integration, neighborhood graph construction (FindNeighbors()) and clustering (FindClusters()) were repeated using the integrated RPCA reduction and the top 30 dimensions. A clustering resolution of 0.5 was used for this analysis. Integrated embeddings for UMAP and *t*-SNE were also recomputed. Cluster identities were renamed accordingly using the RenameIdents() function.

### 2.4. Immunocytochemistry

Immunofluorescent staining was performed on NHCs cultured on glass cover slips placed in a 12-well plate (2000 cells/well) in DMEM supplemented with 1% fetal bovine serum. Cells were fixed with 3% paraformaldehyde (Sigma-Aldrich, St. Louis, MO, USA) for 20 min at room temperature and washed twice with phosphate-buffered saline (PBS). Subsequently, after blocking for 1 h in 5% normal goat serum in PBS (Thermo Fisher Scientific, Waltham, MA, USA), cells were incubated with anti-TREM-2 goat polyclonal antibody for 1.5 h (Invitrogen, Thermo Fisher Scientific, Waltham, MA, USA; cat. no PA5-18763; 1:100 dilution). After three washes in PBS, cover slips were incubated for 1 h at room temperature with Fluorescein (FITC) AffiniPure^®^ Bovine Anti-Goat IgG (H + L) (Jackson ImmunoResearch Laboratories Europe LTD., West Grove, PA, USA, cat. no 805-095-180; 1:500 dilution). Cells were then washed three times with PBS, and cover slips were mounted on slides with VectaShield containing 4′-6-diamidine-2-phenyl indole (DAPI) (Vector Laboratories, Inc., Newark, CA, USA). Negative controls were prepared by omitting the primary antibodies. Images were acquired with a ZEISS Axio Imager Z2 fluorescence microscope equipped with the Zen Pro 2011 acquisition program.

### 2.5. ELISA Analyses

TREM-2 and CD163 serum protein concentrations were measured using a Human TREM-2 ELISA Kit (Catalogue # EH464RB, Invitrogen, Waltham, MA, USA) and Human CD163 ELISA Kit-Quantikine (Catalogue #: DC1630, R&D, Minneapolis, MN, USA) according to the manufacturer’s protocol.

### 2.6. Isolation of RNA and Quantification miRNA and mRNA

For miRNA quantification, total RNA was isolated from 200 µL serum samples and the cultured cells using an miRNeasy Serum/Plasma Advanced Kit (Qiagen, Germantown, MD, USA) and miRNeasy Tissue/Cells Advanced Kits (Qiagen), respectively. cDNA was synthesized using the TaqMan Advanced miRNA cDNA Synthesis Kit (Applied Biosystems, ThermoFisher Scientific, Waltham, MA, USA) according to the manufacturer’s protocol. The levels of miR-34a (478048_mir) and miR-506 (478958_mir), along with miR-16 (477860_mir), which was used as an endogenous control, were measured using TaqMan^®^ Advanced miRNA Assays (Applied Biosystems). Each assay for miRNA expression comprised a 10 μL reaction mixture that contained 5 μL TaqMan^®^ Fast Advanced Master Mix (2×) (Applied Biosystems), 2.5 μL diluted cDNA, 0.5 μL of TaqMan^®^ Advanced miRNA Assay (20×) and 2 μL of RNase-free water.

Total RNA was isolated from the cultured cholangiocytes using an RNeasy Mini kit (Qiagen). RNA was reverse-transcribed into cDNA using a SuperScript IV-First-Strand cDNA Synthesis System Kit (Applied Biosystems) according to the manufacturer’s protocol. qRT-PCR analyses were performed with predesigned TaqMan^®^ Gene Expression Assays: TREM2 (Hs00219132-m1), ADAM17 (Hs01041915-m1) and 18S (Hs99999901-s1) (ThermoFisher Scientific). All TaqMan^®^ MGB probes used spanned exon–exon junctions to exclude genomic DNA as a template in real-time PCR reactions. Each reaction comprised a 10 μL reaction mixture that contained 5 μL of TaqMan^®^ Gene Expression Master Mix (Applied Biosystems), 1.5 μL of diluted cDNA template and 0.5 μL of the probe–primer assay mix. Eukaryotic 18S ribosomal RNA served as an endogenous control.

For all gene expression analyses, the mean cycle threshold values were quantified with Sequence Detection software (version 7500 Software v2.0.2) using Thermo Cycler 7500 Fast real-time PCR (Applied Biosystems). Relative expression levels were determined using the 2^−ΔΔCt^ formula.

### 2.7. Statistics

Graphs were created using GraphPad Prism for Windows version 7.0 (Dotmatics, Boston, MA, USA). All statistical analyses were performed using StatView software (Cary, NC, USA). Comparisons between experimental groups were conducted using a nonparametric Mann–Whitney U test. Paired samples were analyzed using a paired *t*-test. For in vitro experiments, a two-way ANOVA with Fisher’s least significant difference test was applied. All the experiments with cell lines were repeated at least three times on separate occasions. A value of *p* < 0.05 was considered statistically significant.

## 3. Results

**Figure 1:**
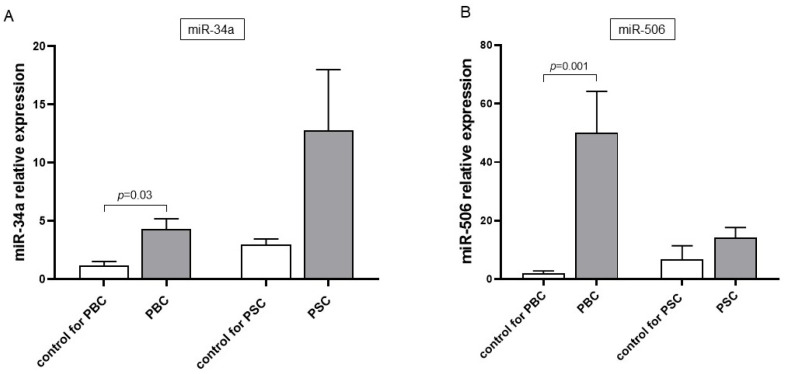
Basal serum expression levels of miR-34a and miR-506 in patients with PBC and PSC before the introduction of UDCA treatment. miR-34a (**A**) expression was significantly elevated in PBC patients compared to age- and sex-matched healthy controls, while no significant difference was observed in PSC patients due to high intra-group variability. miR-506 (**B**) expression was markedly elevated—by several-dozen-fold—in PBC patients relative to controls. Comparisons between experimental groups were performed using the Mann–Whitney U test. Data are presented as mean ± SEM. Abbreviations: PBC, primary biliary cholangitis; PSC, primary sclerosing cholangitis; UDCA, ursodeoxycholic acid.

We examined the basal serum expressions of miR-34a and miR-506 before introducing the UDCA treatment. MiR-34a expression was higher in PBC patients (a 4-fold increase vs. sex-matched healthy subjects (control) for PBC, *p* = 0.03, Figure 1A), but there was no significant difference between PSC patients and the groups of sex-matched controls for PSC. In contrast to PSC patients, we observed significantly enhanced miR-506 expression in the serum of PBC patients (a 25-fold increase vs. PBC controls, *p* = 0.001; Figure 1B).

**Figure 2:**
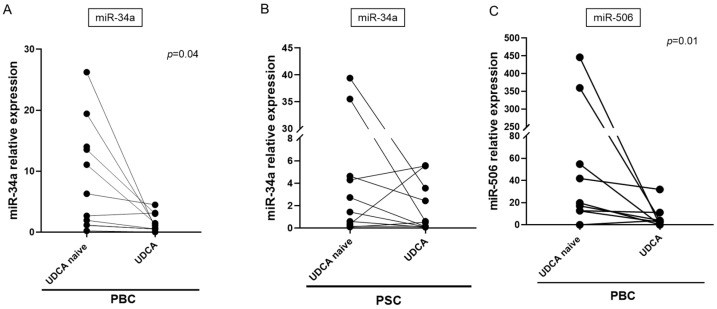
The effect of UDCA treatment on the serum expression of miR-34a and miR-506. UDCA suppressed the expression of miR-34a (**A**) and miR-506 (**C**) in patients with PBC but not PSC (**B**). Each symbol represents one patient. A Student’s paired *t*-test was used to test statistical significance. The median duration of UDCA administration was 44 months (range: 22–56 months). PBC, primary biliary cholangitis; PSC, primary sclerosing cholangitis; UDCA naive, values before the introduction of ursodeoxycholic acid; UDCA, after treatment with UDCA.

It has previously been reported that UDCA can modulate the expression of several miRNAs. Therefore, we analyzed the serum expression levels of miR-34a and miR-506 following UDCA treatment (median duration: 44 months). When the mean microRNA expression in PBC patients treated with UDCA was compared to their pre-treatment levels using ANOVA, we observed that UDCA significantly reduced the expression of miR-34a (mean reduction 75%, *p* < 0.0001) and miR-506 (mean reduction 50%, *p* = 0.02). However, in PSC patients, UDCA had no effect on serum miR-34a levels (Figure 2B). MiR-506 expression was not analyzed in PSC patients after UDCA treatment, as its baseline serum level was not elevated prior to treatment. Additionally, we conducted repeated measures analyses in individual patients before and after UDCA treatment using a Student’s paired *t*-test. The results are presented in Figure 2.

**Figure 3:**
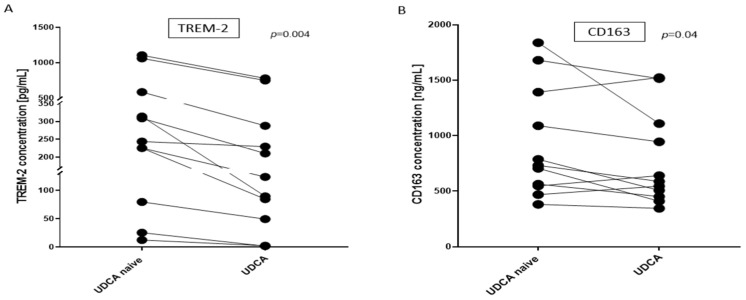
The effect of UDCA treatment on TREM-2 and CD163 in PBC patients. The serum concentrations of TREM-2 (**A**) and CD163 (**B**) were evaluated before (UDCA naïve) and after UDCA treatment (UDCA). UDCA significantly reduced the concentration of TREM-2 and CD163. Each symbol represents one patient. A Student’s paired *t*-test was used to test statistical significance. TREM-2, triggering receptor expressed on myeloid cells 2; UDCA naïve, values before the introduction of ursodeoxycholic acid; UDCA, after treatment with UDCA.

TREM-2 is a known target of miR-34; therefore, we examined whether the UDCA-induced suppression of miR-34 would affect its serum level. Despite miR-34a inhibition after UDCA treatment, we did not observe the up-regulation of TREM-2. Moreover, the introduction of UDCA in PBC patients lowered the serum concentration of TREM-2 (417 ng/mL before UDCA vs. 240 ng/mL after UDCA, *p* = 0.004, Figure 3A). We hypothesized that UDCA treatment can reduce macrophage activation, thus leading to the suppression of TREM-2 concentration. Knowing that sCD163 is a marker of macrophage activation and that its expression is associated with the severity of liver disease, we determined sCD163′s serum concentration. We found that UDCA treatment suppressed the mean serum level values of CD163 for all PBC patient groups (916 ng/mL before UDCA vs. 717 ng/mL after UDCA), and the differences were more pronounced when the values of CD163 concentrations were compared individually for each patient before UDCA vs. after UDCA (paired *t*-test *p* = 0.04; Figure 3B).

**Figure 4:**
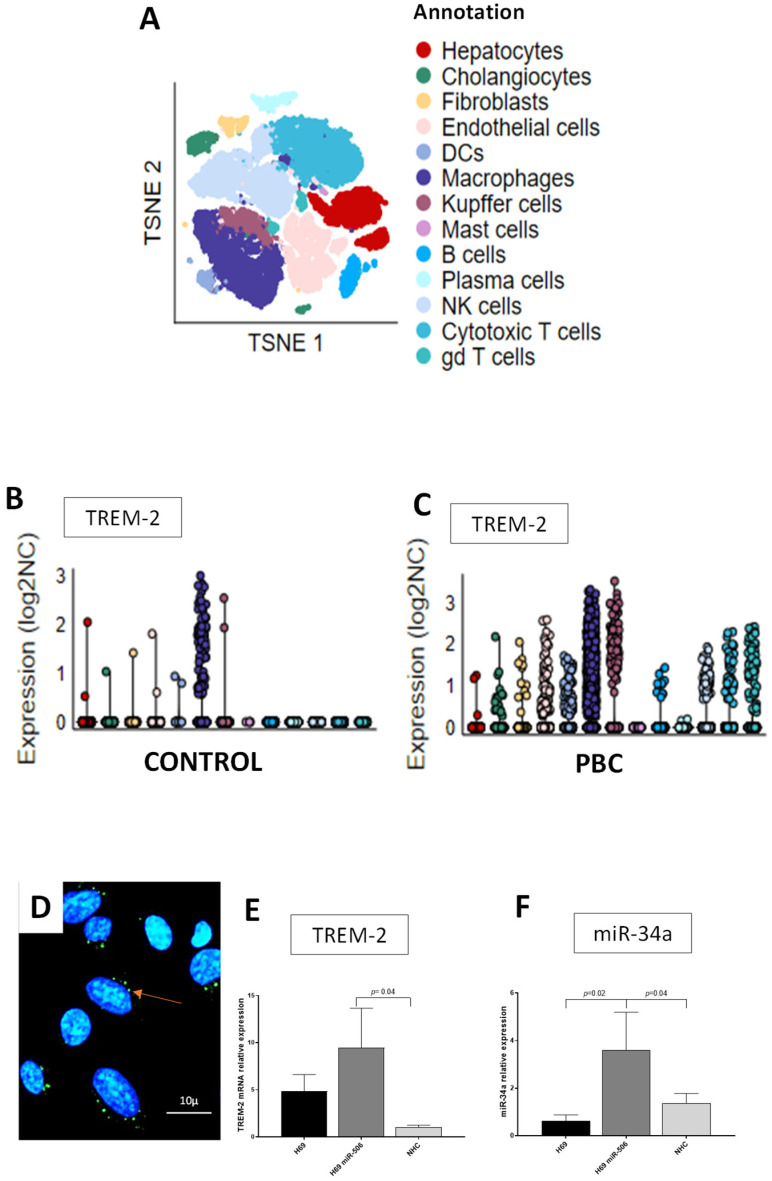
The expression of TREM-2 in the human liver and cholangiocytes. Single-cell RNA sequencing analysis of PBC (*n* = 2) and control (*n* = 5) liver samples. A *t*-SNE plot displaying 13 distinct cell types annotated in the integrated dataset of PBC and control samples (**A**). A jitter plot with the expression of TREM-2 across cell types in control (**B**) and PBC (**C**) groups. The expression of TREM-2 is significantly increased in PBC compared to cells in the control liver. A representative micrograph of NHC shows immunofluorescent staining for TREM-2 proteins (green), while nuclei are stained with DAPI (blue). The red arrow indicates the presence of the TREM-2 protein stained in green (**D**). In comparison to NHC, the baseline expression of miR-34 (**E**) and TREM-2 mRNA (**F**) was the highest in the H69 miR-506 cells. Data are represented as mean ± SEM; *n* = 4. NHC, normal human cholangiocytes; H-69, non-tumor immortalized human cholangiocytes; H69 miR-506 and H69 cells with experimental overexpression of miR-506; PBC, primary biliary cholangitis; TREM-2, triggering receptor expressed on myeloid cells 2.

TREM-2 is a protein primarily expressed by myeloid cells. However, given the role of cholangiocytes in innate and adaptive immune responses, we investigated whether cholangiocytes produce this protein. Single-cell RNA sequencing analysis of the livers of PBC (GSE247128; *n* = 2) and control (GSE115469; *n* = 5) patients revealed that cholangiocytes express TREM-2 (Figure 4A–C), although to a much lesser extent than macrophages and KCs. Furthermore, the presence of TREM-2 proteins in NHC cells was confirmed via immunofluorescence staining (Figure 4D). We next examined whether TREM-2 mRNA and miR-34a were expressed in three human cholangiocyte cell lines: a primary culture of normal human cholangiocytes (NHCs), as well as non-tumor immortalized human cholangiocytes (H69) with or without experimental overexpression of miR-506 (PBC human model). Our data showed that human cholangiocytes express TREM-2, with the highest level observed in H69-miR-506 cells compared to both controls, i.e., H69 (3.5-fold increase vs. H69, *p* = 0.02; Figure 4E) and NHC (9-fold increase vs. NHC, *p* = 0.04; Figure 4E). Similarly, the baseline expression of miR-34 was 3.5-fold higher in H69 miR-506 than in H69 or NHC cells (*p* = 0.02, *p* = 0.04, respectively; Figure 4F).

**Figure 5:**
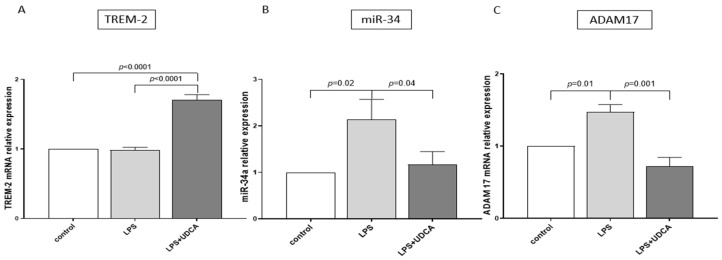
Effects of UDCA on LPS-induced expression of TREM-2, miR-34a, and ADAM17 in NHC cell lines. qPCR analysis demonstrated that TREM-2 (**A**) was not induced by LPS stimulation alone; however, co-treatment with UDCA resulted in activation of this gene. The LPS-induced expression of miR-34a (**B**) and ADAM17 (**C**) mRNA was significantly suppressed by co-treatment with UDCA. Data are presented as mean ± SEM from four independent experiments. Statistical analysis was performed using two-way ANOVA followed by Fisher’s least significant difference (LSD) test. Abbreviations: ADAM17, a disintegrin and metalloprotease 17; LPS, lipopolysaccharide from Escherichia coli; NHC, normal human cholangiocytes; TREM-2, triggering receptor expressed on myeloid cells 2; UDCA, ursodeoxycholic acid.

The extracellular domain of TREM-2 is shed by proteases such as ADAM17, which leads to the generation of sTREM2. Previous studies have shown that UDCA affects ADAM17 expression. Therefore, we analyzed the effect of UDCA on TREM-2, miR-34a and ADAM17 expressions in NHC after LPS stimulation. We found that the LPS-induced inflammatory response did not affect TREM-2 expression (Figure 5A). However, the application of UDCA in these cells did enhance TREM-2 expression (a 1.7-fold increase vs. LPS or controls, both *p* < 0.001; Figure 5A). Furthermore, LPS incubation of NHC led to the induction of both miR-34 (2-fold, *p* = 0.02 vs. control) and ADAM17 (1.5-fold, *p* = 0.01 vs. control), which was blocked by co-treatment with UDCA (*p* = 0.04 and *p* = 0.001 vs. LPS, respectively; Figure 5B,C).

## 4. Discussion

UDCA is the first-line treatment for patients with PBC, but its effectiveness varies from patient to patient, and the molecular mechanisms triggering the therapeutic effects of UDCA are not yet fully understood. In the present study, we attempted to identify the effect of UDCA on the serum expression of miRNAs, which are known to be significantly up-regulated in PBC patients, namely miR-34 and miR-506. Additionally, the observed effect of UDCA was verified in vitro in cholangiocyte cell lines.

To the best of our knowledge, the profile of miR-34a and miR-506 expression in patients before the introduction of UDCA treatment has never been previously examined. In this study, we showed that the baseline expression of miR-34a before the introduction of UDCA treatment was higher in the serum of patients who were diagnosed with PBC compared to healthy controls. Previously, the enhanced expression of miR-34a has been reported in PBMCs [13] and in the livers of PBC patients [36]. However, those analyses were conducted after the introduction of UDCA treatment. The observed up-regulation of miR-34a in PBMCs was associated with an increased expression of genes involved in fibrogenesis and the epithelial–mesenchymal transition (EMT) through the TGF-β1/smad pathway [13]. Our study clearly showed that UDCA treatment decreased the serum level of miR-34a in cholestatic PBC patients, which may potentially slow down disease progression by inhibiting fibrosis and EMT. Hence, UDCA effectively modulated the miR-34a/SIRT/p53 pathway. In rat primary hepatocyte cell lines, UDCA causes the suppression of proapoptotic miR-34a/SIRT1/p53 signaling [37]. Likewise, in a group of morbidly obese patients, a short-term UDCA treatment decreased miR-34a expression in vesicle-free serum but not in liver tissue [38]. We did not observe any changes in the serum expression of one of the miR-34a target genes, i.e., SIRT1, after UDCA treatment in PBC patients, which is consistent with observations in NAFLD patients [38].

Another well-known target of miR-34 is the anti-inflammatory receptor TREM-2, which diminishes TLR-mediated signaling. Inflammation is an important factor in the progression of cholestatic diseases, and the role of different components of the gut–liver axis has been recently highlighted [23]. Previously, we described the up-regulation of TREM-2 in the livers of patients with PBC and PSC, which positively correlated with markers of disease progression [21]. Moreover, Trem-2 expression was found to be noticeably higher in nonparenchymal liver cells, including KCs and activated HSCs in the liver of murine models of obstructive cholestasis [21]. In this study, suppressed miR-34 expression in response to UDCA treatment did not lead to the induction of TREM-2, and a significant suppression of this receptor was noticed. We assumed that the observed TREM-2 down-regulation in response to UDCA treatment was due to the suppression of TNF-α and IL-6 production and promotion of the anti-inflammatory stage in macrophages. Given that sCD163 is a marker of macrophage activation, and its serum concentration correlates with liver inflammation and response to UDCA treatment [32], we evaluated levels of CD163 in the serum of our patients. Our investigation showed that the high levels of CD163 were lowered by the UDCA treatment in PBC patients. Thus, we assume that the suppressed level of TREM-2 may be an indirect result of the anti-inflammatory effect of UDCA.

Given that UDCA is a bile acid that is passively taken up in the large intestine and transported through the bile ducts lined by cholangiocytes, we investigated the effect of UDCA exposure in cholangiocytes, i.e., cells that have direct contact with UDCA. Cholangiocytes express TLRs and participate in innate and adaptive immune responses. Although TREM-2 is mainly produced in myeloid cells, we investigated whether cholangiocytes also express certain TREM-2 levels. Our study confirmed the expression of TREM-2 in cholangiocytes via single-cell RNA sequencing of liver tissue and via immunofluorescence staining of NHC cells. The up-regulation of TREM-2 mRNA and miR-34a was observed in H69 cholangiocytes with miR506 overexpression. MiR-506 causes multiple PBC-like features in biliary epithelial cells, and pro-inflammatory cytokines enhance the expression of miR-506 [16]. Therefore, to investigate how NHCs react in a pro-inflammatory environment, we analyzed the expression of genes involved in liver inflammation. We assumed that miR-34a, TREM-2 and ADAM17 are potential points of UDCA action. LPS stimulation of normal cholangiocytes led to the induction of miR-34a and ADAM17, which has not been previously reported for this type of cell. Previously, it was suggested that the pro-inflammatory environment promotes ADAM17 activation only in immune cells [18]. However, our study showed that the overexpression of miR-34a and ADAM17 was suppressed by UDCA treatment. Interestingly, the expression of TREM-2, which suppresses pro-inflammatory signaling, was unaffected by LPS stimulation but was significantly induced by UDCA treatment. Additionally, this in vitro study demonstrated that UDCA application resulted in the inhibition of ADAM17 expression, which may lead to the reduction in sTREM-2 levels. ADAM17 is believed to destroy the surface domain of TREM-2, and the effectiveness of ADAM-17 inhibitors in stabilizing TREM-2 at the cell surface has been shown [39]. We postulated that ADAM17 was inactivated by UDCA treatment, and, therefore, the serum level of sTREM-2 was reduced, which is in line with a previous report [25].

Consistent with earlier studies, which showed enhanced miR-506 expression in cholangiocytes of PBC patients and in explanted liver tissue from patients with end-stage PBC compared to healthy controls [9,16], we also observed the overexpression of miR-506 in PBC patients. However, it is important to highlight that our study is the first to demonstrate increased serum levels of miR-506. Moreover, our study is novel in that we analyzed serum miR-506 levels in newly diagnosed PBC patients with no prior UDCA treatment. We showed that UDCA therapy significantly reduced serum miR-506 levels, a finding not previously reported. MiR-506 promotes PBC-like features in cholangiocytes by down-regulating the Cl/HCO_3_ anion exchanger 2 (AE2) gene and InsP3R3, leading to cholestasis, cellular stress and apoptosis [16]. It also induces PDC-E2 overexpression and activates immune responses, contributing to the autoimmune phenotype characteristic of PBC [35]. Thus, the observed inhibition of miR-506 by UDCA may have an additional therapeutic effect by reducing AE2 inhibition.

## 5. Conclusions

In conclusion, we confirmed that the introduction of UDCA treatment decreased the expression of miR-34a, miR-506 and sCD163 in the serum of PBC patients, which may lead to the suppression of pro-inflammatory signaling and result in the reduction of serum TREM-2 levels. Additionally, the application of UDCA in normal cholangiocyte cell lines abolished the LPS-induced increase in miR-34a and ADAM17 expression. This prospective study provides additional information on the therapeutic potential of UDCA as a miRNA modulator in cholestatic liver diseases.

## Figures and Tables

**Table 1:** Demographic and laboratory data of the study cohorts.

Parameter	PBC			PSC		
	Before UDCA (*n* = 27)	After UDCA (*n* = 11)	*p* Before vs. After	Before UDCA (*n* = 17)	After UDCA (*n* = 10)	*p* Before vs. After
Gender (Male/Female)	2/25	1/10	0.8	11/6	7/3	0.7
Age (years)	54 ± 11	58 ± 9	NS	36 ± 10	42 ± 9	NS
GGTP (IU/L, NR male < 60, female < 35)	287 ± 59	98 ± 16	0.003	498 ± 104	259 ± 78	0.007
Bilirubin (mg/dL, NR 0.2–1.1)	1.40 ± 0.5	0.77 ± 0.2	0.23	2.0 ± 0.8	0.67 ± 0.1	0.14
ALP (IU/L, NR 30–120)	240 ± 25	148 ± 18	0.0006	329 ± 69	289 ± 71	0.17
AST (IU/L, NR 5–35)	99 ± 37	41 ± 6	0.11	128 ± 53	55 ± 9	0.18

Abbreviations: ALP, alkaline phosphatase; AST, aspartate aminotransferase; GGTP, gamma glutamyl transferase; NR, normal range; PBC, primary biliary cholangitis; PSC, primary sclerosing cholangitis; UDCA, ursodeoxycholic acid.

**Table 2:** Demographic and laboratory data of the control cohorts.

Parameter	Control for PSC (*n* = 9)	Control for PBC (*n* = 16)	*p* PBC vs. PSC
Gender (Female/Male)	2/7	15/1	0.0002
Age (years)	35 ± 1.2	52 ± 3.7	0.004
GGTP (IU/I, NR male < 60, female < 35)	WNR	WNR	
Bilirubin (mg/dL, NR: 0.2–1.1)	WNR	WNR	
ALP (IU/L, NR 0.2–1.1)	WNR	WNR	
AST (IU/L, NR 5–35)	WNR	WNR	

Abbreviations: ALP, alkaline phosphatase; AST, aspartate aminotransferase; GGTP, gamma-glutamyl transpeptidase; PBC, primary biliary cholangitis; PSC, primary sclerosing cholangitis; WNR, within normal range.

## Data Availability

The data presented in this study are available on request from the corresponding author.
